# Community intervention to reduce social isolation in older adults in disadvantaged urban areas: study protocol for a mixed methods multi-approach evaluation

**DOI:** 10.1186/s12877-019-1055-9

**Published:** 2019-02-18

**Authors:** María José López, Carolina Lapena, Alba Sánchez, Xavier Continente, Ana Fernández, the Evaluation Group of the “Schools of Health for older people”, Alba Sánchez, Anna Fernández, Anna Pérez, Camila Astorga, Carme Cortina, Carolina Lapena, Cristina Rey, Elia Diez, Enriqueta Pujol, Ferran Daban, Gemma Castillo, Iolanda Robles, Lucía Artazcoz, María José López, Mariona Pons, Marc Marí-Dell’Olmo, Montse Petit, Natalia Sagarra, Nuria Calzada, Olga Juárez, Xavier Bartoll, Xavier Continente

**Affiliations:** 1 Public Health Agency of Barcelona, Lesseps, 1, 08023 Barcelona, Spain; 2 Spanish Consortium for Research on Epidemiology and Public Health (CIBERESP), Madrid, Spain; 3 Sant Pau Institute of Biomedical Research (IIB Sant Pau), Barcelona, Spain; 4 Centre d’Atenció Primària Sanllehy, Gerència d’Àmbit d’Atenció Primària Barcelona Ciutat, Institut Català de la Salut, Barcelona, Spain; 5 Unitat de Suport a la Recerca Barcelona Ciutat, Institut Universitari d’Investigació en Atenció Primària Jordi Gol (IDIAP Jordi Gol), Barcelona, Spain; 6 Universitat Autònoma de Barcelona, Bellaterra, Cerdanyola del Vallès Spain; 7 Preventive Medicine Department, University Hospital of Bellvitge, Hospitalet de Llobregat, Barcelona, Spain

**Keywords:** Social isolation, Aging, Older people, Evaluation

## Abstract

**Background:**

The proportion of older people has dramatically increased in recent decades. Moreover, social and demographic trends show a global increase of older people at risk of loneliness and lack of social relationships. The objective of this study was to evaluate the process, the effectiveness and the cost-effectiveness of a planned 22 weekly group sessions called School of Health for Older People to reduce social isolation.

**Methods:**

This is a mixed methods multi-approach evaluation that includes: 1) A qualitative evaluation among coordinators and participants taking part in the intervention, through in depth-interviews and focus groups, respectively. The main topics covered will be positive and negative aspects of the intervention, suggestions for its improvement, opinions on different aspects of the intervention, and perceived benefits; 2) A quantitative quasi-experimental design, comparing a group of individuals taking part in the intervention with another group with similar characteristics not receiving the intervention. Data will be collected at the beginning and at the end of the intervention. Social support will be measured through questions drawn from the Medical Outcomes Study and the National Social Life, Health, and Aging Project questionnaires. Psychological morbidity will be measured through Goldberg’s General Health Questionnaire, and Health-related Quality of Life will be measured through the EuroQoL questionnaire. Information on visits to the primary care center in the years before and after the intervention will be obtained from the electronic records of the primary care centers; 3) A cost-utility analysis, which will be conducted from a health system (primary care) perspective, including direct costs of the program and the primary care health services used. The effects of the intervention will be measured on quality-adjusted life years.

**Discussion:**

There is an urgent need for studies assessing the effectiveness and the efficiency of potential interventions to reduce social isolation among older persons. The results of this study will help to fill the knowledge gap in this area and might be especially useful for the development of social and public health policies and programs for older people in disadvantaged neighborhoods in urban areas.

**Trial registration:**

NCT03142048 retrospectively registered (April 11, 2017).

## Background

The proportion of older people has dramatically increased in recent decades [1]. In the city of Barcelona, 21.2% of the population is 65 years or older, and projections indicate that this percentage will increase to 23.9% in 2031 [2].

Social and demographic trends show a global increase of older people at risk of loneliness and lack of social relationships. This may be due to certain aging-related factors such as retirement or the loss of a partner or close friends [3]. Previous studies have found higher rates of loneliness in deprived urban areas [4]. Furthermore, other studies have found that some of the variables significantly associated with loneliness in older adults included poor income and lower educational level [5], and that living in a deprived area adds barriers to social engagement [6]. Overall, recent studies confirm that rural residents reported less social isolation and more social relationships than urban residents [7]. Therefore, disadvantaged urban areas need to be studied, and evaluated interventions in these areas should be prioritized.

Social relations are associated with good mental health, while their absence is linked to a significant increase in morbidity and mortality [8–10]. Because of its high prevalence and the evidence of its impact on health and wellbeing, social isolation is an important public health issue.

Based on the aforementioned evidence, interventions are needed to reduce social isolation and its negative effects on quality of life. Although preventing and addressing social isolation in older people is a priority in health policies, there is a clear lack of evidence on the effectiveness of health promotion activities in this field, as well as wide heterogeneity in the interventions and their quality [11–15].Therefore, studies evaluating the effectiveness of interventions designed to reduce the impact of social isolation on wellbeing and quality of life in older people are much needed. Detailed protocols on how to evaluate this complex interventions can help to standardize the evaluation across interventions and homogenize the evidence on this area. In this sense, this protocol might be useful as a tool for future interventions to be evaluated.

Since 2008, an intervention called the “Schools of Health for Older People” has been implemented in some deprived neighborhoods of Barcelona. This intervention is part of the comprehensive action *Health in the neighborhoods* [16], focused on reducing health inequalities in the city of Barcelona by implementing community interventions in the most deprived neighborhoods. The main goal of the “Schools of Health for Older People” is to reduce social isolation in this collective, as well as its potential harmful health effects. As shown in numerous studies [17, 18], these effects can be especially important in low-income older people. The main objective of this protocol is to describe the mixed methods multi-approach evaluation study designed to assess this intervention.

## Hypothesis

The intervention evaluated in this protocol will significantly improve social support, mental health, general health status and distinct dimensions of quality of life in the intervention group (IG) compared with the comparison group (CG). Among participants in the IG, the intervention will also significantly reduce the number of visits to their primary care centers. Furthermore, this intervention will be cost effective.

## Objectives

### General objective

To evaluate the process, effectiveness and cost-effectiveness of an intervention to reduce social isolation and its consequent negative impact on health of older people.

### Specific objectives

1. To evaluate the implementation process of the intervention (participant profile, positive and negative aspects of the intervention, barriers and facilitators in its implementation, quality of the intervention, and satisfaction).
2. To assess the impact of the intervention on social support, self-perceived health status, mental health and quality of life among participants, and visits to the primary care center.
3. To determine the cost-effectiveness of the intervention.

## Methods

### Study design

This is a mixed methods multi-approach evaluation that includes:

- a qualitative evaluation among coordinators and participants who underwent the intervention through in-depth interviews and focus groups, respectively;
- a quantitative quasi-experimental design, comparing a group of individuals taking part in the intervention (IG) with another group with similar characteristics not receiving the intervention (CG). Data will be collected at the beginning and at the end of the intervention;
- an economic evaluation, which includes a cost-utility analysis.

### Study setting and participants

#### Qualitative evaluation

The study population will include coordinators of the School of Health (community nurses from the Public Health Agency of Barcelona) and participants who will attend the School of Health in the intervention neighborhoods. In-depth interviews will be carried out with the coordinator of each IG, who will be selected through intentional opinatic sampling (non-randomized sampling in which the researcher selects a sample based on their knowledge about the study and population) [19].

School of Health participants will be adults aged 65 years or older residing in two disadvantaged neighborhoods of Barcelona (Spain) where the School of Health will be implemented. Theoretical sampling will be designed to select participants from the School of Health. For each neighborhood, two groups of participants will be selected according to their risk of experiencing loneliness. The risk of loneliness will be measured through various questions extracted from the National Social Life, Health, and Aging Project (NSHAP) study [20], included in the questionnaire used in the quantitative study. Individuals who report feeling a lack of companionship often, regardless of their cohabitation status (with relatives or alone), will be categorized as ‘at high risk’ and those reporting hardly ever feeling alone and living with relatives will be classified as ‘low risk’. Thus, four focus groups (two per neighborhood) with 6 to 10 assistants will be assembled.

For each focus group, we will select participants providing the widest range of profiles to ensure a variety of discourses according to age, sex, marital status and educational level. Community nurses will be asked about the ability of the selected participants to follow the dynamics of a focus group. Then, the selected participants will be contacted by telephone, informed about the study and asked if they are willing to participate. Those refusing to take part in the study will be replaced by other individuals with similar characteristics. Those who accept to take part in the focus group will have to sign an informed consent form.

#### Quantitative quasi-experimental design

The study population will consist of adults aged 65 years or older residing in the disadvantaged neighborhoods of Barcelona (Spain) selected for the study. The intervention neighborhoods (*n* = 2) and comparison neighborhoods (n = 2) will be selected on a convenience basis, ensuring similar socioeconomic characteristics, including the percentage of people with primary studies or less, unemployment rates and disposable household income rates.

The intervention will be offered to adults aged 65 years or older, living in one of the two selected neighborhoods, and recruited through primary care centers, social services and civic centers for older people. Once individuals have accepted to participate, there will be a pre-registration process that will record their names and telephone numbers. Later, the participants will be contacted by telephone to arrange an appointment, in which the person will be informed individually about the study. After agreement, he/she will sign the informed consent form and complete a questionnaire.

To calculate the sample size, we took into account that 45% of people aged 65 years or more in Barcelona reports fair or poor health [21] and assumed that the intervention would reduce this prevalence by 17 points. Accepting an alpha risk of 0.05% and a power of 80% in a unilateral contrast, and assuming a loss of 10%, we estimated that a theoretical sample of 79 participants in the IG and 79 participants in the CG will be needed.

We expect to recruit 80 individuals in the IG and 80 in the CG. To have similar groups, the recruitment settings (primary care centers, social services and civic centers) would be taken into account, by selecting the same percentage of people from each setting.

The exclusion criteria are the following:

- Participants with difficulties in maintaining participation for 6 months.
- Participants with difficulties in understanding or expressing themselves in Spanish or Catalan.

#### Economic evaluation

The study population and the selection criteria will be the same as those in the quantitative quasi-experimental design.

### Participant timeline

The participants will be contacted by telephone to arrange and appointment, in with the person will be informed individually about the study. After agreement, he/she will sign the informed consent form and complete the baseline questionnaire (December–January 2015). Those participants not able to arrange an appointment, will receive the consent form and complete the baseline questionnaire during the first session of the intervention (just before starting). The intervention will last 22 weeks (January–June 2015). At the end of the last session the participants will complete the follow-up questionnaire (June 2015). Regarding the qualitative analysis, the in-depth interviews and focus groups will be carried out between 6 to 9 months after the intervention (January–March 2016).

**Fig. 1:**
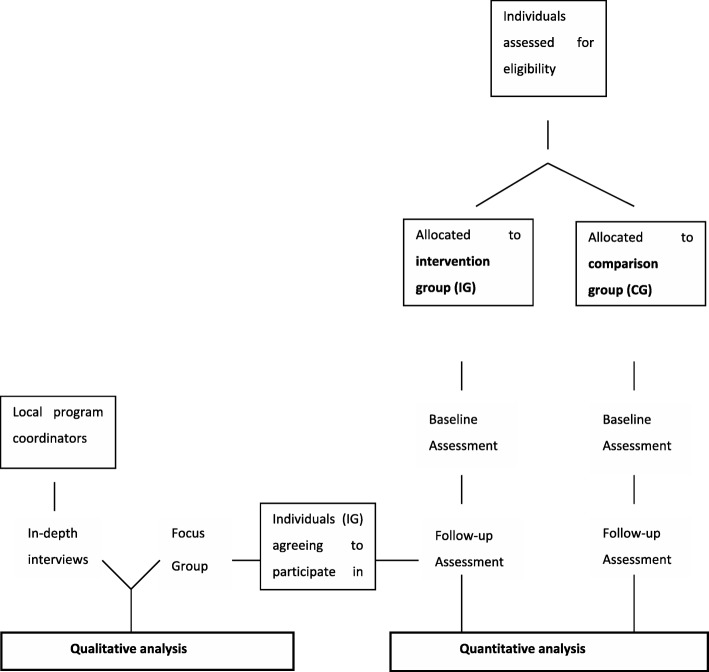
Flow chart of enrolment, allocation and follow-up

The participant flowchart of the study procedure is outlined in Fig. 1.

### Description of the intervention

The intervention “School of Health for Older People” consists of 22 weekly group sessions of 1.5 h each, in which issues related to health—including both biological and psychological issues—and social topics, are discussed. Attendance is free and the sessions will be held in community centers of the selected neighborhoods. In addition to helping participants learn about different health issues, the intervention encourages interaction among participants and works on skills in different fields, such as nutrition, management of emotions, self-medication, sexuality or physical activity. Furthermore, some sessions included visits to public spaces of the neighborhood and leisure activities adapted to older people.

**Fig. 2:**
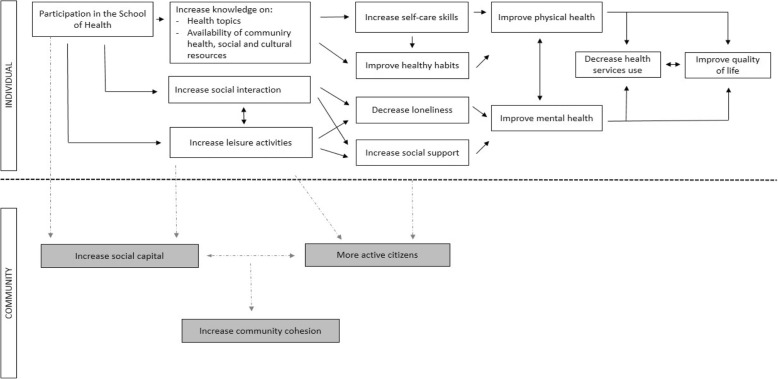
Explanatory model of the potential effects of the “Schools of Health for Older People”

Furthermore, most sessions are led by professionals who are experts on the topic covered and work in the neighborhood (professionals from the health services, social services, markets or neighborhood associations), making it easier to inform participants of the neighborhood’s available resources. The aim of the intervention is to decrease social isolation and loneliness and, therefore, to improve mental health, self-perceived health and wellbeing (see the explanatory model of the potential effects of the intervention in Fig. 2).

A brief description of the contents of one “School of Health for Older People” is shown in Table 1.

**Table 1:** Example of the contents of sessions in the “School of Health for Older People”

Session 1	Introduction and group instructions. Colds and flu
Session 2	Adequate nutrition and fluid intake
Session 3	Elimination of body waste. Hygiene
Session 4	Laughter therapy
Session 5	Health hazards in the household and first aid
Session 6	Self-medication: the less the better
Session 7	Sexuality: enjoying it at any age
Session 8	Preventing scams and thefts
Session 9	Circus workshop
Session 10	Going out: how to do it in the safest way
Session 11	Memory and the five senses
Session 12	Emotions
Session 13	Physical activity at the park
Session 14	The value of older age
Session 15	How to become a volunteer at our neighborhood
Session 16	Our household remedies: let’s share!
Session 17	Diarrhea and Vomiting: what to do?
Session 18	Heat waves
Session 19	Songs of our times. Farewell

### Outcome measures

#### Qualitative evaluation

The main topics covered in the qualitative evaluation will be positive and negative aspects of the intervention, suggestions for its improvement, opinions on different session contents and logistic aspects of the intervention, and perceived benefits derived from the intervention.

#### Quantitative quasi-experimental design

##### Main outcomes

Social support will be measured through questions taken from the Medical Outcomes Study: Measures of Quality of Life Core Survey (MOS) [22] and NSHAP questionnaires [20]. Psychological morbidity will be measured through Goldberg’s General Health Questionnaire (GHQ-12) [23], and health-related quality of life will be measured through the EuroQoL (EQ-5D) questionnaire [24]. Objective information will be obtained on visits to the primary care center during the year before and after the intervention through access to the electronic records of the primary care centers.

All these variables will be measured simultaneously in individuals in the IG and CG. Data will be collected through baseline and post-intervention questionnaires administered by trained researchers.

##### Intervention-related variables

We will register attendance of participants at each session in the IG. Additionally, at the end of the School of Health, we will measure participants’ satisfaction with questions such as “please rate your general satisfaction with the School of Health from 0 to 10;”“ please rate your satisfaction with the speakers, place/space, frequency, schedule, and length of sessions from 0 to 10” “ Would you recommend the School of Health to a friend? ” These questions will be included in the post-intervention questionnaire.

##### Sociodemographic variables

Sociodemographic information will only be collected in the baseline questionnaire including the following variables: age, sex, marital status (single/ married/ divorced/ widowed), cohabitation status (living alone/living with someone else), educational level (illiterate /primary / secondary / university) and residential neighborhood.

#### Economic evaluation

The economic evaluation will be conducted from a health system (primary care) perspective, including direct costs of the program and the primary care health services used. The time frame will be 6 months and consequently no discounting will be applied to the calculations.

##### Costs

The costs of the program will be calculated based on the hourly wages of the public health professionals involved in the design and implementation of the intervention, obtained from the standard professional wages in the Official Gazette of the Catalan Government; the wages of the community nurses involved in the coordination and development of the workshops, obtained from the Catalan Institute of Health retributions report; and the costs of the venues and materials used throughout the intervention. The costs of the primary care medical and nurse visits either at the health center or at the patient’s home will be drawn from the Official Gazette of the Catalan Government on healthcare costs for the last published year.

##### Effects

The effects of the intervention will be measured in quality adjusted life years (QALY), through the EQ-5D questionnaire. Spanish tariffs will be used to estimate the utility of health states described by the participants. QALYs [25] will be assessed at baseline and at 6 months (end of the intervention), and QALYs will be calculated by multiplying utility by the amount of time a patient spent in a particular health state. Linear interpolation will be used for transitions between health states. QALYs will be assessed at baseline and at 6, 12 and 18 months.

### Data analysis

#### Qualitative evaluation

A thematic analysis will be carried out with the support of ATLAS.ti.software. In-depth-interviews and focus groups will be recorded and transcribed literally. Interviewers will keep a diary in which any reaction to events occurring during the research will be recorded.

The analysis will combine the inductive and deductive definition of codes. Preanalytical intuitions will be formulated after successive readings of the transcriptions and the observation notes. Next, three multidisciplinary investigators will create an initial analytical plan based on the most relevant topics (codification). The creation of categories by grouping the codes will be based on the criterion of similarity in relation to the objectives of the study and the emerging elements.

#### Quantitative quasi-experimental design

A descriptive analysis will be conducted to rule out significant differences between baseline characteristics (sociodemographic variables and outcomes studied) in the IG and the CG. The chi-square test will be used to compare qualitative variables. Continuous variables with normal distribution will be compared using the Student t test, and the Mann-Whitney U test will be used in non-normally distributed variables. In case of differences, a further multivariate effectiveness analysis will be conducted adjusting for these variables.

Further descriptive analyses will be conducted for results at baseline and at follow-up. Categorical outcomes will be described through percentages, and continuous outcomes will be described through mean scores and standard deviations or the median and interquartile range, as required. Differences between pre- and post-intervention measurements will be assessed and compared using the McNemar test, paired t-test, or sign test, according to the type of variable and distribution.

To assess the effectiveness of the intervention, a two-sample McNemar test will first be conducted to analyze differences in changes in pre- and post-intervention results between the IG and the CG for each main outcome. In those outcomes showing differences between the groups at the baseline measurement, Poisson regression models with robust variance will be built. In each model, the independent variables will be the group (IG or CG), the pre-intervention measurements for the outcome assessed and any relevant characteristics that differ between the groups at the baseline assessment.

#### Economic evaluation

The incremental cost-utility ratio (ICUR) will be calculated by subtracting the costs of the CG from the costs of the IG and dividing the result by the QALYs for the CG subtracted from the QALYs for the IG. The incremental costs and the incremental utility will be modeled by generalized linear models, taking into account the distribution of both costs and effects.

To estimate the uncertainty related to the ICUR, we will calculate 95% confidence intervals for the mean cost differences using the non-parametric confidence interval. Bootstrapping with 1000 replications will be performed.

Bootstrapped cost effects pairs will be plotted on cost-effectiveness planes and used to estimate cost-utility acceptability curves (CUACs). In the cost-utility planes, the ‘x’ axis represents the difference in QALYs and the ‘y’ axis the difference in costs. CUACs demonstrate the probability that an intervention is cost effective at a specific ceiling ratio, which is the amount of money society is willing to pay to gain one extra unit of effect. Willingness to pay values will range from 0€ to 50,000€ [26].

The robustness of the estimates will be addressed by conducting sensitivity analyses. This analysis will comprise a variation in the unit cost of the primary care medical or nurse visits, in the costs of the workshop leaders’ hourly wage and in the venue rental cost.

### Ethics considerations

Participants will be informed both verbally and in writing about the aims, methods, procedures and measures performed during the study. They will be also informed about ethics issues such as confidentiality, their right to ask any questions during the study, and their right to withdraw at any time without penalty. To ensure that all participants have received proper information about the study and have agreed to participate, all participants will be asked to sign a written consent form. The research team is committed to performing this study in accordance with the Good Clinical Practice Guidelines of the Declaration of Helsinki. This protocol was approved by the *Comité Ético de Investigación Clínica Parc de Salut Mar (code* n° 2015/6500/I).

## Discussion

### Strengths and limitations

A potential limitation of the study is the length of follow-up, since the post-intervention measurements will be carried out immediately after the end of the intervention (6 months). Therefore, we will not be able to assess its long-term effects. Another limitation of the quantitative part of the study is that, because the design is quasi-experimental, there may be some differences between the IG and the CG. However, a statistical comparison of the main sociodemographic variables will be assessed and if any differences are found, they will be taken into account in the adjusted regression models.

A strength of the study is that, as far as we know, it is the first to assess the effectiveness and cost-effectiveness of an intervention focused on reducing social isolation in older people in deprived neighborhoods in an urban environment. Importantly, this is a multi-approach evaluation assessing the process, effectiveness and cost-effectiveness of an intervention while using mixed methods (quantitative and qualitative). Furthermore, the tests applied to measure the main outcomes (social support, psychological morbidity and health-related quality of life) are validated tests that not only allow for assessment of the potential impact of the intervention but also for comparison with other studies.

### Implications

Aging is one of the greatest social and economic challenges for European societies. At the same time as cities are growing, their share of older residents is increasing. By 2025, more than 20% of European citizens will be 65 years or older [27]. Ensuring the integration of older people in society is essential to promote the wellbeing of older urban residents. Therefore, studies assessing the effectiveness and efficiency of potential interventions to reduce the social isolation of older people are urgently needed.

The results of this study will help to fill the knowledge gap in this area and might be especially useful for the development of social and public health policies and programs for older people in disadvantaged neighborhoods of urban areas.

## Abbreviations

CG: Comparison group
CUAC: Cost-utility acceptability curves
EQ-5: EuroQoL-5D
GHQ-12: Goldberg’s General Health Questionnaire
IG: Intervention group
ICUR: Incremental Cost-Utility Ratio
MOS: Measures of Quality of Life Core Survey
NSHAP: National Social Life, Health, and Aging Project
QALY: Quality Adjusted Life Years
